# Individual identification of inbred medaka based on characteristic melanophore spot patterns on the head

**DOI:** 10.1038/s41598-023-27386-w

**Published:** 2023-01-12

**Authors:** Hajime Morizumi, Naozo Sugimoto, Tomohiro Ueno

**Affiliations:** Human Health Sciences, Graduate School of Medicine, Kyoto University, Kyoto, Japan

**Keywords:** Medaka, Translational research

## Abstract

With disease progression, individual differences appear, even in an animal disease model with genetic homogeneity. Therefore, non-invasive long term observation and individual identification is desirable for late-onset diseases. To this end, the natural markings used in ecological studies are preferable to the external invasive markings used in animal husbandry and fisheries management. Here, we propose using the distribution pattern of melanophore spots on the head of an inbred strain of medaka, a small fish model organism with monotonous pigmentation, as biometric identifier. Long term and variation analyses show different patterns whose characteristics can be attributed to individual animals. These findings were also valid in a non-inbred medaka strain and will help individual follow-up of late-onset disease medaka models for the elucidation of the pathogenesis and drug discovery.

## Introduction

Medaka (*Oryzias latipes*) is a small freshwater fish with a short generation time and extra-uterine development of numerous transparent eggs. Medaka’s inbreeding tolerance has allowed the generation of many inbred strains, which helped sequencing a draft of the genome^1^. An inbred strain is defined after > 20 sequential generations of sibling mating^2,3^ and by being homozygous at > 98.7% of all gene loci^4^. By 1996, some inbred medaka strains were created by > 75 sequential generations of sibling mating^5^. In addition, large-scale genetic screens have been performed^6,7^. Due to these characteristics, medaka has been used as a model organism in genetics and developmental biology^8–10^. With the development of genome editing technology^11^, various human disease models, such as a p53 knockout model^12^ and a Parkinson’s disease model^13^, have been generated. Among these disease models, long term observation of adult individuals is preferred for late-onset diseases such as neurological and neurodegenerative diseases, and drug discovery for drugs with a long administration period. Due to complex mechanisms and stochastic onset, however, rather large individual differences have been found in disease progression in laboratory animals otherwise genetically homogeneous and controlled rearing environment^14,15^. Therefore, non-invasive individual long term tracking of disease progression is desirable and will be possible with appropriate individual identification.

Individual identification by external markings, such as ear tags, tattooing, and branding, has been long applied in animal husbandry for livestock traceability^16,17^. Moreover, in fisheries, external markings, such as a coded wire tag, have been used for individual identification^18^. In these cases, handling easiness of artificial markings has been a priority so long as it does not hinder livestock and fish growth. On the other hand, in ecological and behavioral studies, individual identification focuses on not affecting animals' nature, since the main goal is observing their intact behavior and interactions with other individuals and the environment. In this sense, photographing natural markings, such as zebra stripes^19^ or a whale’s tail shape^20^, is an appropriate method. Natural markings are inherent to individuals, and as such do not affect the target state nor is it disturbing like attaching and reading artificial markings on captured wild animals^21^ and can be utilized in animal biometrics. Since genetically diverse populations are studied in ecological and behavioral research, individual variations in natural markings are large enough to distinguish individuals. On the other hand, in laboratory animals used as disease models, genetic and environmental homogeneity is important^22^. Therefore, large individual variation of natural markings may not occur in such animals. However, isolation of a laboratory animal may change its behavior and growth. Despite these potential issues, the ear blood vessel pattern of an inbred mouse was used as natural marking. However, for measurements, the mouse was anesthetized and its ear was pressed on the prototype device^23^. In small fish, due to its poikilothermic nature and small body size, it is preferable to avoid direct touch. Inbred medaka are similar to inbred mice in inbreeding cycles and show only monotonous pigmentation like the mouse fur. On the other hand, in zebrafish, which is another small fish model organism, a characteristic stripe pattern on its body can be utilized as natural marking. In addition, despite a high level of genetic homogeneity (5% polymorphism), only 16 sequential generations of sibling mating have been achieved^24^, possibly with larger genetic diversity than that of inbred medaka.

In this study, we aimed to utilize the distribution pattern of melanophore spots on medaka’s head as natural marking for individual identification, since its location and number appears to vary from individual to individual. In addition, as the melanophore spots are visible even to the naked eye, its distribution pattern can be easily recorded in a non-invasive medaka. Using an inbred medaka strain, we verified that the distribution pattern is individual, and that it does not change over time. To confirm the utility of the method, we applied it to the individual identification of non-inbred medaka.

## Results

### Long term verification of a distribution pattern of melanophore spots

**Figure 1:**
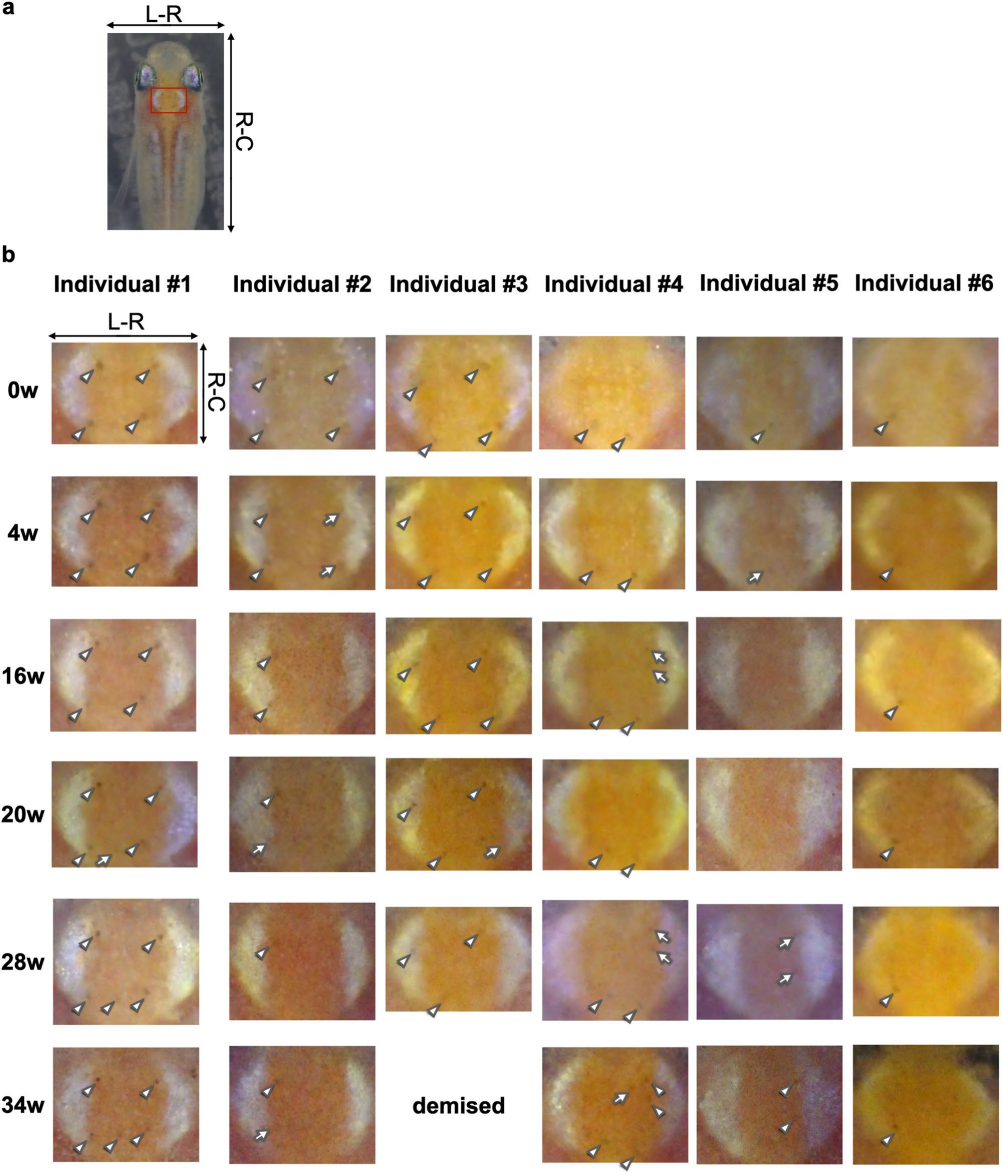
Extracted position of a mesencephalic region and its enlarged images of a mesencephalic region of six adult inbred medaka consecutively acquired at six time points. (**a**) Representative position of a mesencephalic region in the whole body. The extracted mesencephalic region (Individual #1, 0w) is shown as a red rectangle. (**b**) Enlarged images of an extracted mesencephalic region. Each column corresponds to individual medaka (Individual #1–#6), and each row to a single time point (0, 4, 16, 20, 28, and 34 weeks after the first acquisition). Individual #3 was demised at the last time point. A white arrowhead points toward a discerned melanophore spot. A white arrow indicates an appearance change of a melanophore spot between two consecutive time points. The Hd-rR strain (F97) was used as inbred medaka. Individuals #3, #4, and #6 were male, and Individuals #1, #2, and #5 were female. R-C: rostral-caudal axis, L-R: left-right axis.

We acquired head images of six adult inbred medaka of the Hd-rR strain (F97, 97 sequential generations of sibling mating) at six time points over 34 weeks (0, 4, 16, 20, 28, 34 weeks) to obtain a characteristic distribution pattern of melanophore spots. Figure 1a shows a representative position of a mesencephalic region, and Fig. 1b shows six sets of enlarged extracted mesencephalic region; discerned melanophore spots are indicated by white arrowheads and arrows. At the first time point (week 0), Individual #1 had four melanophore spots near the four corners of two white crescent-like regions forming a square-shaped pattern, Individual #2 had four melanophore spots forming a trapezoid-shaped pattern, Individual #3 also had four melanophore spots forming a parallelogram-shaped pattern, Individual #4 had only two melanophore spots forming a horizontal line between the bottom of two white regions, Individual #5 had just one melanophore spot in the bottom center, and Individual #6 also had one melanophore spot but at the bottom left. Each individual had its own characteristic pattern by which it could be distinguished. At 4 weeks, Individuals #1–#6 had the same number of discerned melanophore spots at the same positions forming the same distribution patterns. At 16 weeks, however, two melanophore spots on the right-hand side of Individual #2 and one at the bottom of Individual #5 were missing (white arrows in the second row of Fig. 1b, 4 w). On the other hand, two new vertically aligned spots could be observed next to the upper part of the right white region of Individual #4 (white arrows in the third row of Fig. 1b, 16 w). Despite missing and appearing spots, the remaining spots maintained their original positions. Even if the original distribution pattern had changed at this time point, we could still distinguish each individual from another, and attribute each distribution pattern to each individual comparing this and previous time points. Similarly, there were missing and appearing melanophore spots at the fourth, fifth, and sixth time points (20, 28 and 34 weeks in total), but Individuals #1–6 were identifiable through the remaining distribution patterns. In addition, at four-week intervals, such as between 1st–2nd and 3rd–4th time points, the changes in the distribution pattern were smaller than at longer intervals. Note that one melanophore spot on the bottom left of Individual #2 and two vertically aligned spots at the upper right side of Individual #4 disappeared at one time point and reappeared at the next time point.

### Variation and stability of the distribution pattern of melanophore spots

**Figure 2:**
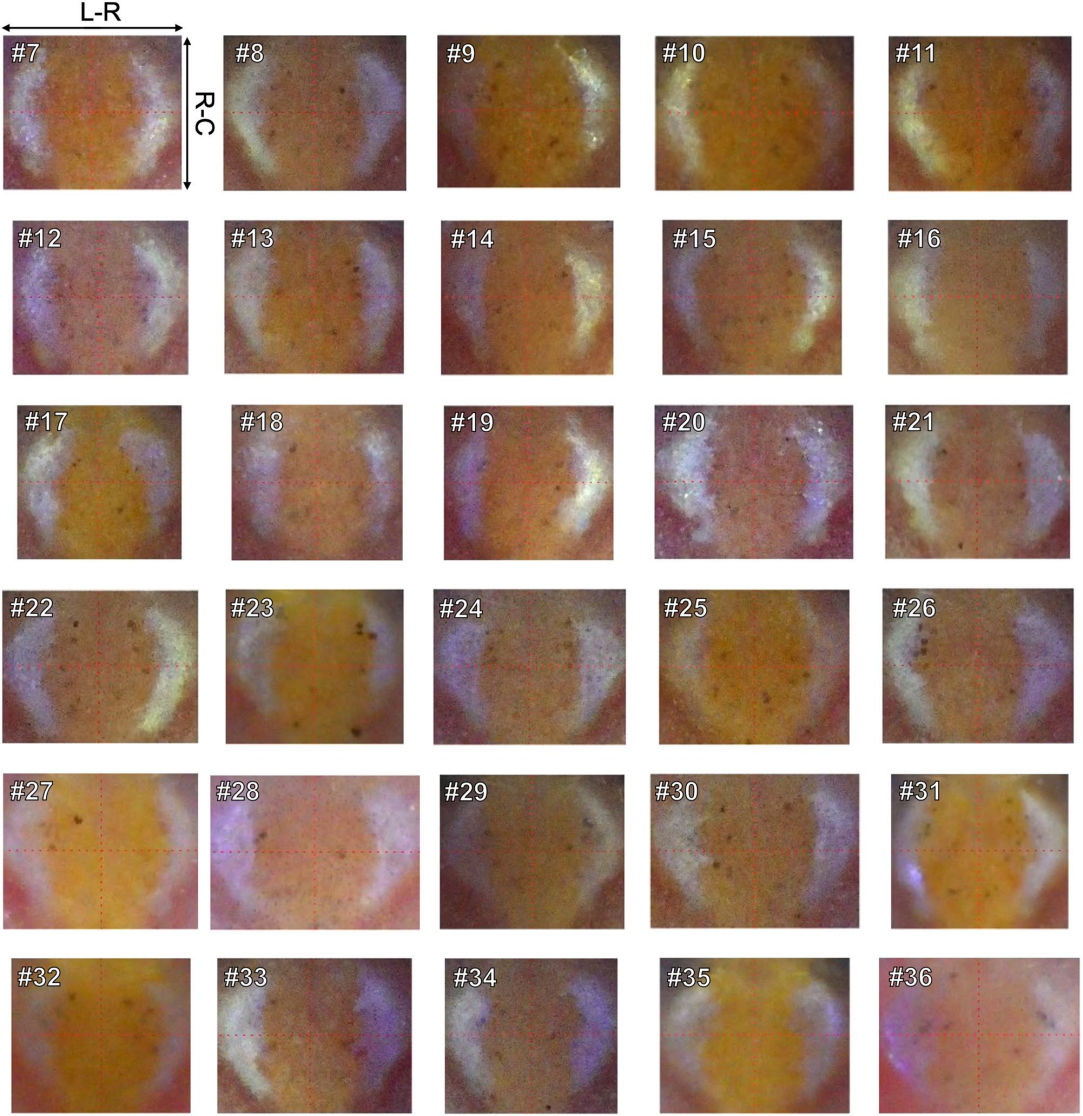
Individual distribution patterns of melanophore spots of 30 adult inbred medaka at the first time point. A number in each image corresponds to an individual identifier. The red dotted lines serve a guide. The Hd-rR strain (F97) was used as inbred medaka. Individuals #7, #9, #10–#13, #16, #17, #23, #25, #27, #29, #31, #32, and #35 were male, and Individuals #8, #14, #15, #18–#22, #24, #26, #28, #30, #33, #34, and #36 were female. R-C: rostral-caudal axis, L-R: left-right axis.

We obtained 30 individual distribution patterns of adult inbred medaka of the Hd-rR strain (F97) twice at a 4-week interval. We increased the number of individuals from 6 to 30 to examine whether the distribution pattern showed enough variation. The 4-week interval was chosen to limit changes in individual distribution pattern. Figure 2 shows 30 distribution patterns of melanophore spots at the first time point (Individuals #7–#36). The number of melanophore spots was not limited to < 5, there were 10 in Individual #8 and 9 in Individual #13. In addition, various distribution patterns could be discerned, such as a fanning-like shape in Individual #13, a square-with-a-right-triangle-at-its-upper-right-vertex-like shape in Individual #23 and an isosceles-triangle-like shape next to the left white region of Individual #27. Since they were all different, we could distinguish the distribution patterns in Fig. 2.

**Figure 3:**
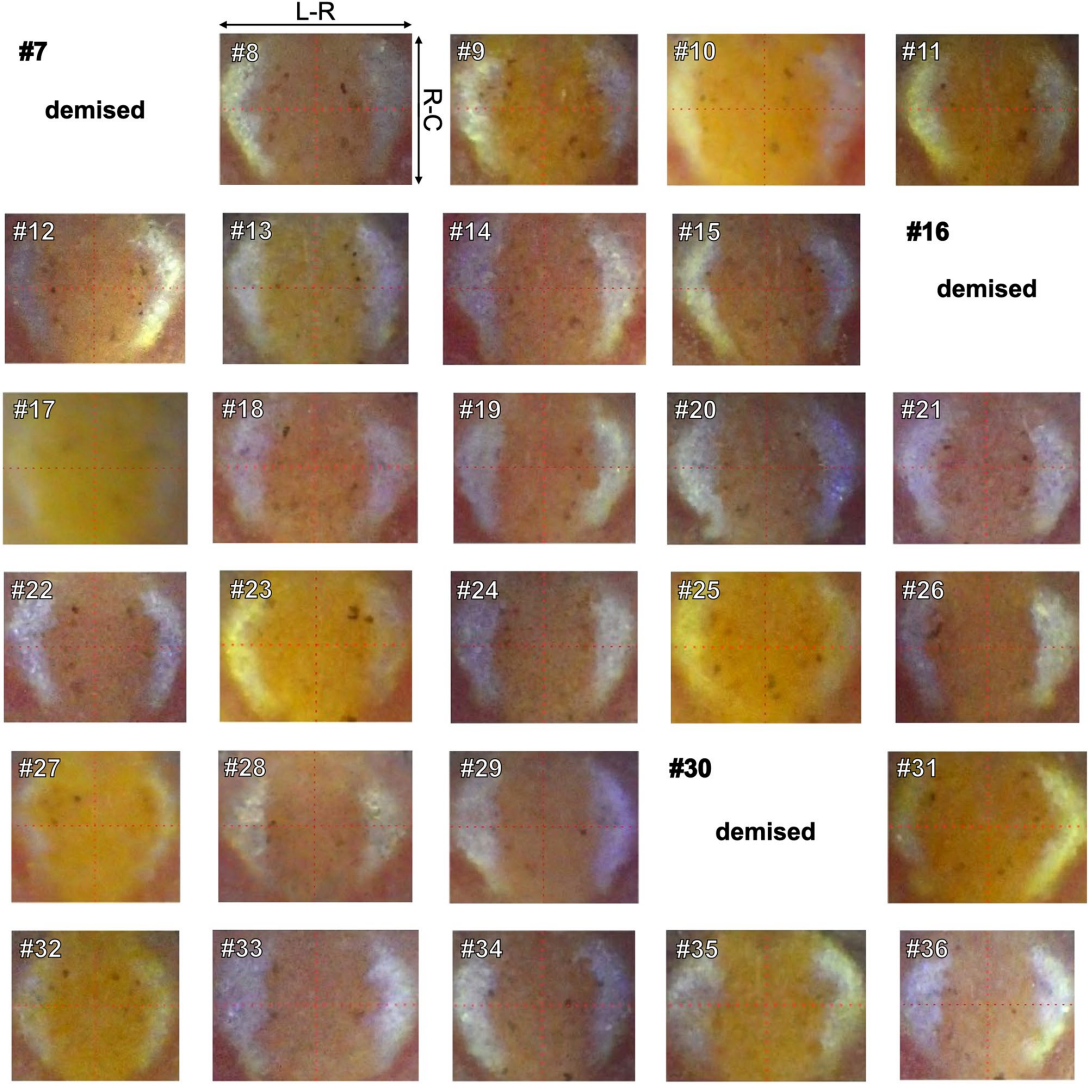
Individual distribution patterns of melanophore spots at the second time point. These distribution patterns were acquired four weeks after the first acquisition (Fig. 2). Three medaka (#7, #16, and #30) were demised by this time. Individual #17 was infected with Matsukasa disease. The same individual identifiers are used as in Fig. 2. The red dotted lines serve a guide. R-C: rostral-caudal axis, L-R: left-right axis.

At four weeks, three medaka had died. Figure 3 shows the remaining 27 distribution patterns shown in the same numerical order as in Fig. 2. One of the remaining medaka (Individual #17) was infected with Matsukasa disease, and treated as demised. Although the appearance of melanophore spots was slightly different from that at the first time point, we could still differentiate the distribution patterns. For example, in Individual #8 melanophore spots formed a two-vertical-lines-shape. A similar shape could be found in Individual #13, but the left line was thicker at the top and the right line closer to the white region. Furthermore, the distribution patterns showed the same characteristics as at the first time point. We could discern the fanning-like shape, the square-with-a-right-triangle-at-its-upper-right-vertex-like shape and the isosceles-triangle-like shape in Individuals #13, #23, and #27, respectively.

### Verification of a non-inbred strain case

**Figure 4:**
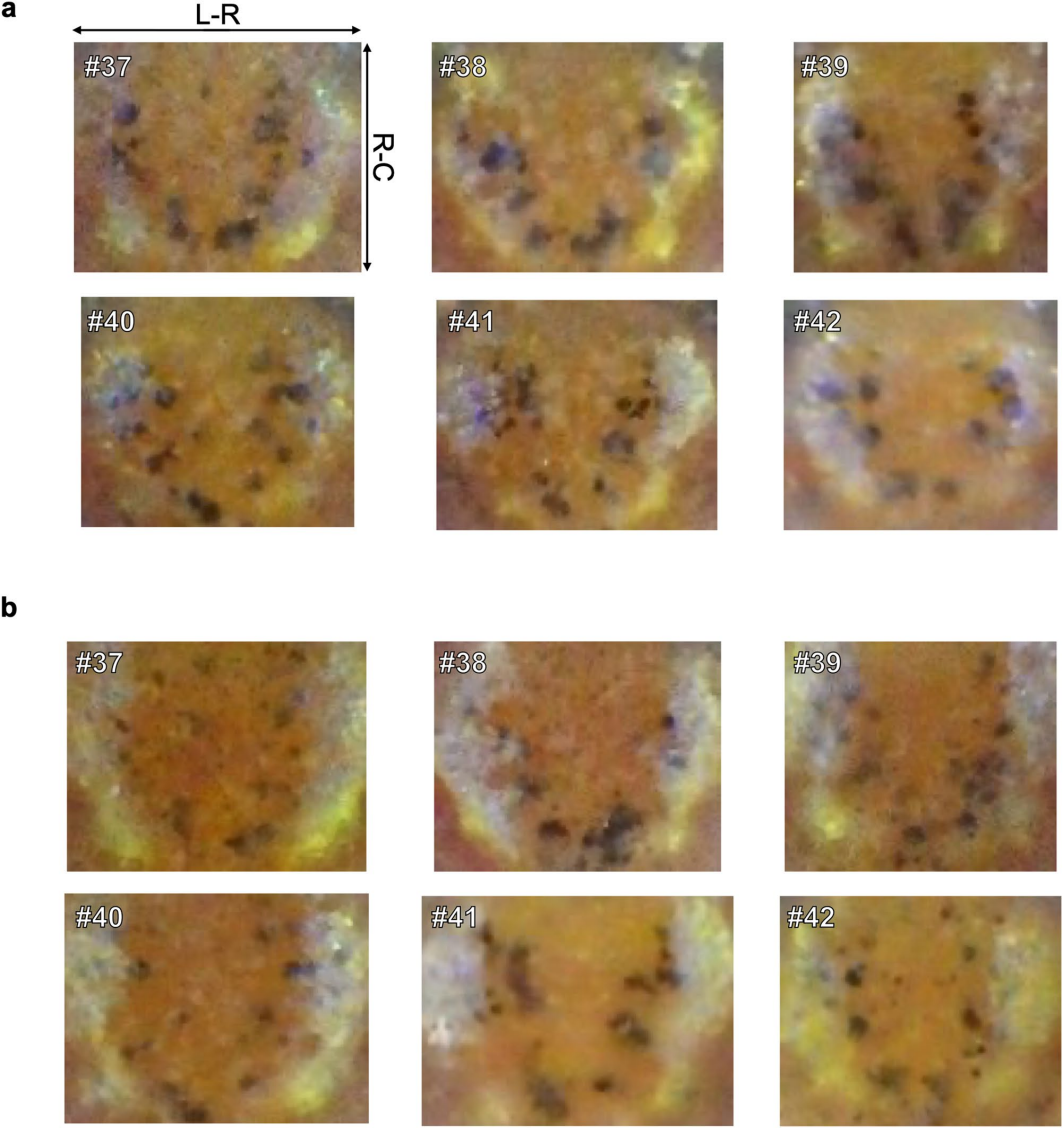
Individual distribution patterns of melanophore spots of six young non-inbred medaka at two consecutive time points. Distribution patterns at the first (**a**) and second (**b**) time point, separated by four weeks. A number is utilized as an individual identifier in each image. The Kyoto-Cab strain was used as non-inbred medaka. Individuals #38, #40, and #41 were male, and Individuals ##37, #39, and #42 were female. R-C: rostral-caudal axis, L-R: left–right axis.

In addition to inbred strains, we observed six individual distribution patterns of young non-inbred medaka of the Kyoto-Cab strain twice at a 4-week interval. The distribution patterns at the first time point (Individuals #37–#42) are shown in Fig. 4a. Comparing with those of inbred fishes, there were larger melanophore spots, some did not have a circular shape and looked merged. At first glance, all distribution patterns appeared to form a bowl-like shape, but close examination revealed different patterns. In Individuals #37 and #40, the bowl-like shapes had some melanophore spots inside, but at a higher position in Individual #37, at a lower position in Individual #40. In Individuals #38 and #42, the shape was rounded at the bottom; shallower in Individual #38, deeper in Individual #42. In Individuals #39 and #41, the shape bottom was sharper, but the shape itself was tilted in Individual #39, and other spots were clustered at the top left in Individual #41. Figure 4b shows six distribution patterns 4 weeks later with the same numerical individual marks as before. The melanophore spots became smaller and the distance between spots larger than at the first time point. Despite these changes, we could differentiate between the six distribution patterns using the same variations of the bowl-like shape obtained at the first time point.

### Blind identification tests

To verify the usefulness of the distribution pattern of melanophore spots as individual identifier, blind pairing tests were performed using the distribution pattern in Figs. 2, 3, 4. Three examinees were instructed to match two randomized sets of images taken at the first (Figs. 2, 4a) and second time point (Fig. 3, 4b) separately for inbred and non-inbred strains (Supplementary Files 1, 2). The examinees were blinded to the numerical identifiers in Figs. 2, 3, 4. The results of the pairing tests are shown in Table 1. In the inbred strain test, all examinees could pair all images of healthy medaka, leaving out those of demised and infected fish. On the other hand, in the non-inbred strain test, Examinee 1 and 2 had a 100% identification rate, but Examinee 3 confused Individual #39 in Fig. 4a with Individual #42 in Fig. 4b and vice versa resulting in a 67% identification rate.

**Table 1:** Results of blind identification tests.

	Inbred	Non-inbred
Examinee 1	26/26 (100%) (demised and infected: 4/4)	6/6 (100%)
Examinee 2	26/26 (100%) (demised and infected: 4/4)	6/6 (100%)
Examinee 3	26/26 (100%) (demised and infected: 4/4)	4/6 (67%)

## Discussion

In this study, we acquired a head image of individual inbred and non-inbred medaka and extracted a distribution pattern of melanophore spots on a mesencephalic region over time. Comparing the patterns of the six inbred medaka, we found different distribution patterns over 34 weeks (Fig. 1b). Although part of the pattern changed gradually in some individuals, we could attribute each pattern to each individual (Fig. 1b). Then, we expanded the analysis to 30 individuals and found that all distribution patterns could be distinguished (Fig. 2) and that pattern characteristics were maintained after four weeks (Fig. 3). Moreover, in the blind identification test, all three examinees showed 100% correct answer rates (Table 1). From these findings, we concluded that individual inbred medaka could be identified over a long time period based on the characteristics of its pattern of melanophore spots. In addition, in contrast to work in wild animals, in laboratory animals we can determine the time interval for image acquisition at will.

The appearance of the melanophore spots on the mesencephalic region changed over time in the same individual (Figs. 1, 2, 3). Melanophore spot recognition, however, was affected by image quality (Supplementary Figs. 1, 2), so not-discernible spots in an image do not mean nonexistent melanophore spots. Although the image quality varied during the long observation period, it would be reasonable to assume that a melanophore spot discerned at two consecutive time points was real. The case of vanishing and reemerging in Fig. 1b, since it only vanished at one time point, this could be an artifact due to the image quality. In Fig. 1b, four different time intervals (4, 6, 8 and 12 weeks) were used. At those time intervals, the appearance change was largest at 12 weeks and almost none at 4 weeks. Although the time interval was not short enough to determine whether the melanophore spot itself appeared suddenly or gradually, the distribution pattern as a whole changed gradually depending on interval length; therefore, the probability of changing pattern was minimized in a time window of 4 weeks. In fact, the observed change in distribution pattern in Figs. 2, 3 was small enough to connect the pattern at the second time point with that at the first time point. Therefore, we could conclude that a 4-week interval was short enough for individual identification of adult inbred medaka.

We increased the number of medaka from 6 to 30 for the variation and stability verification test (Figs. 2, 3) since according to the central limit theorem in probability theory, the mean of random samples tends to be normally distributed when the number of samples is ≥ 30 even if the random sample itself does not follow a normal distribution^25^. Therefore, we utilized 30 medaka to investigate the distribution pattern, although we did not compute the arithmetic mean and statistical significance of the derived properties. Moreover, in most tank systems as well as ours, considering the tank size and fish density, medaka would be maintained as < 30 individuals in a tank, so verifying the patterns in 30 medaka would show enough robustness in the individual identification based on the distribution pattern against the individual variation in ordinary rearing conditions^26^.

In non-inbred medaka, we also found six distinguishable distribution patterns with overall constant characteristics after four weeks (Fig. 4). At the first time point in the non-inbred strain case, medaka fish were 10 weeks of age, corresponding to a rapid body-growth period. By 15 weeks, medaka’s body size has increased substantially and since sexual maturity has been reached, the growth rate slows down^27^. During the rapid body-growth period, the distance between melanophore spots would increase faster and changes in distance and direction might not be homogeneous. Therefore, the larger changes observed in the non-inbred strain were probably due to the larger growth rate between time points.

In addition to the larger change in the distribution pattern between time points, larger size and number of melanophore spots were found in the non-inbred strain than those in the inbred strain. The non-inbred Kyoto-Cab strain has not only a larger genetic diversity but also a different genotype from the inbred Hd-rR strain. The body coloration of medaka is determined by chromatophores in the skin and a melanophore is one of them^26^. The observed melanophore spot can consist of a single or multiple melanophores and its size can depend on the number of melanophores and/or quantity of pigments in a melanophore. As a melanophore-related gene, *slc45a2* was found^28^. The Kyoto-Cab strain has the *B’* allele in the *slc45a2* locus causing a variegated pigmentation pattern; on the other hand, the Hd-rR strain has the *b* allele showing defective melanin phenotype^26^. To reveal which of the genetic heterogeneity and the genotype difference affects more the observed variation of the distribution pattern in the two strains, comparisons between a non-inbred strain with the *b* allele such as d-rR and an inbred strain with the *B’* allele such as HNCMH2^26,29^ are required.

**Figure 5:**
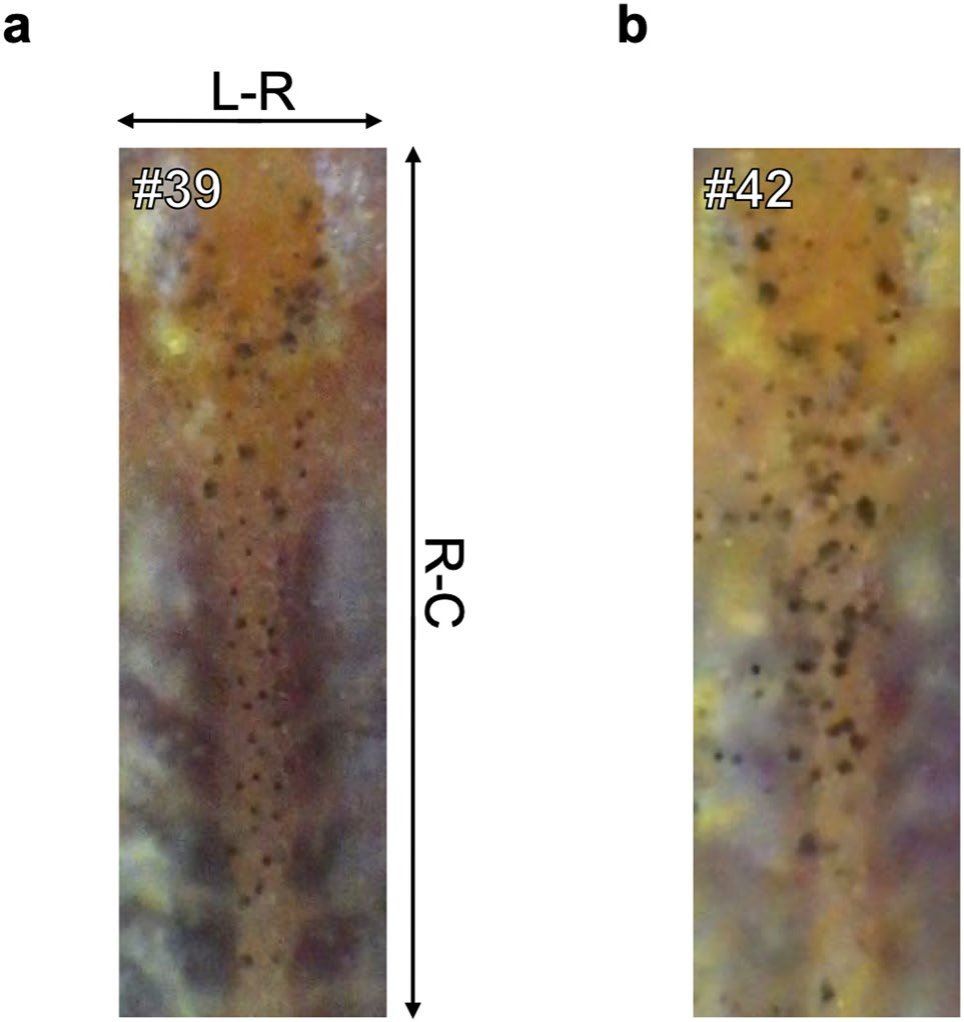
Extended views of melanophore spot distribution patterns on the dorsal side. Distribution patterns from the mesencephalic to the mid spinal cord region of Individual #39 (**a**) and #42 (**b**) in Fig. 4b are shown. The mesencephalic regions of the patterns are the same as Fig. 4b #39 and #42. R–C: rostral-caudal axis, L-R: left–right axis.

In the blind identification test of non-inbred medaka, two examinees showed 100% correct answer rate but one examinee only 67% (Table 1); however, this result confirmed individual identification even in non-inbred medaka. In the test, Examinee 3 failed in the identification of Individuals #39 and #42, in which the size of melanophore spots changed between time points. Since the melanophore spot patterns maintained the same characteristics, for individual identification it would be better to pay attention to individual melanophore spots and the overall pattern. Nevertheless, a large number of melanophore spots and fast growth rate would make difficult tracing individuals by their distribution patterns. Thus, a shorter interval of image acquisition might be better. In addition, the patterns in other body parts such as the dorsal side of the body just above the spinal cord could be utilized as supplementary information (Fig. 5). Despite the difficulty of focusing on a wider region, the broader distribution pattern of the melanophore spots might help tracking individuals. Moreover, a guide in extracted images of the mesencephalic region might help recognizing a characteristic pattern. In the enlarged image of inbred medaka, a vertical and horizontal line guide were placed at the automatically-chosen center (Figs. 2, 3, Supplementary File 1). In the blind test of non-inbred medaka, however, the guide was removed (Fig. 4, Supplementary File 2), suggesting that this guide helped perceive the relative position of the melanophore spots, even if not at the same anatomical position in both two time points.

There are other inbred medaka strains with the same *b* allele in the *slc45a2* locus apart from the Hd-rR strain used in this study, such as HO4C and AA2^30^. Given differences in craniofacial morphology between inbred medaka strains derived from different origins^31^, the distribution patterns of melanophore spots in other inbred medaka strains can differ from the ones in this study. Although a large and small number of melanophore spots were investigated in this study, verification in other inbred strains is needed. On the other hand, the genetic variation within the same inbred strain can be estimated as well. In this study, the F97 of the inbred strain was utilized. At F97, the probability of heterozygote occurrence can be calculated at 1.06 × 10^−9^. Considering that the genomic size of medaka is 700.4 Mb^1^ and assuming that all variations come from the heterozygote occurrence, individual variation in the genome of the same inbred strain can be estimated to exist in 0.74 bases (700.4 × 10^6^ × 1.06 × 10^−9^). The estimated ~ 0.74 base number may represent a single-nucleotide polymorphism in the medaka genome. Although the existence of single-nucleotide polymorphism changes might increase susceptibility to a disease^32^, future research is required to unveil the causes for the observed individual variations of the distribution pattern of melanophore spots in the inbred strain.

Besides genetic heterogeneity and genotypic differences, a sex difference may affect the distribution pattern of melanophore spots. Especially, an increase in the number of melanophores and xanthophores has been reported in males during the breeding season, known as nuptial coloration^33^. In this study, the ratio of male to female medaka was 1:1. Except that Examinee 3 failed to identify two female medaka (Individuals #39 and #42) in the blind test, we could not find clear indication of the sex difference in stability, variation, and appearance of the distribution pattern. Another factor affecting the body coloration of medaka is a breeding environment. Morphological color change can occur to adapt the environment^34^. In addition, pigments can be taken from the environment^26^. In this study, the fish tank environment was kept so that algae on the tank surface did not grow to the level of blocking out light from the tank surroundings. Although different algae condition existed in each tank, no clear dependence on the tank surface condition was found in stability, variation, and appearance of the distribution pattern. In a shorter time scale, such as within 5 min, we found that tank bottom color and lighting environment did not affect the discerning of melanophore spots (Supplementary Figs. 1, 2). However, to know acceptable stability and variation of the tank surface condition, further research is needed.

Besides the Hd-rR and the Kyoto-Cab strains, wild type, HNI, and Kaga strains are also used in many laboratories^26,30^. These wild type and wild type-originated strains have the *B* allele in the *slc45a2* locus and show more black pigmentation phenotype. As found in the non-inbred Kyoto-Cab strain case, more melanophore spots could make more difficult to distinguish the characteristics of the distribution pattern. Therefore, quantitative evaluation based on binary data as described below would be more important. In addition, it would be possible to utilize characteristics of a melanophore spot region pattern formed by multiple melanophore spots as biometric identifier, instead of the distribution pattern of melanophore spots.

Although artificial tagging is an invasive individual identification method, less invasive methods exist such as passive integrated transponder (PIT) tagging^35,36^ and visible implanted elastomer (VIE) tagging^36^. While application of the PIT tagging has been limited to a slightly larger fish^36^, the VIE tagging has already been used with small fish (< 100 mm)^37,38^. Especially, VIE tagging has shown higher marker visibility and stability than the those by proposed method. However, requirements of anesthesia and injections would affect the survival rate depending on species, body length, and proficiency of the investigator^38^. Moreover, in zebrafish, VIE tagging affects social preferences^39^. Since body coloration of medaka affects mating behaviors^40^, VIE tags might also affect medaka social behaviors. In contrast, our proposed method could exclude any unknown possibility of effects on the fish survival rate and social behaviors and is easily available to any laboratory with a commercially available digital compact camera and a computer.

In this study, a digital compact camera with digital microscopic mode was used to capture an enlarged view of medaka’s mesencephalic region. To increase image quality, a stereomicroscope with a digital camera could be utilized, although it would involve higher cost and possible need for anesthesia. In addition, qualitative measures rely on recognition of distribution pattern characteristics. However, the qualitative measures might induce human error, especially applying to a distribution pattern with a faint melanophore spot. Thus, in addition to the current window level adjustment, a mesencephalic image could be binarized, thereby using an appropriate threshold. Furthermore, using a binarized distribution image and a pattern recognition method in machine learning^41^ might allow quantifying characteristics and automate identification. It might also lead to more precise identification and studies in the original environment, as in ecological studies. Furthermore, in blind identification tests, a one-to-many identification could be performed only in the beginning. Since all images of individual medaka at both time points were available, the method of elimination could be employed. In laboratory animals this is not a problem, since similar conditions to those in this study would be used. In addition, individuals with difficult distribution patterns to distinguish could be separately maintained after the initial observation.

Despite the potential for future improvements to the current set-up, our proposed method of individual identification based on the distribution pattern of melanophore spots would serve as animal biometrics relevant to medical and neurological research in medaka. In a time-series analysis, the proposed method would make it possible to return medaka to its original rearing environment after one experiment without imposing any burden. Therefore, it can be used in the long term observation needed for research on neurological and neurodegenerative diseases and on drugs with a long administration period.

In conclusion, we showed that the distribution pattern of melanophore spots on the head of inbred and non-inbred medaka differed in each individual and maintained its characteristics over time. In blind identification tests, three examinees scored high correct answer rates; thus, in the typical rearing environment, this pattern, recorded at 4-week intervals for adult and shorter time intervals for young medaka, could be utilized as biometric identifier, and play an important role in long term observations in medical and neurological studies.

## Methods

### Ethics statement

All procedures were in accordance with national guidelines and approved by the institutional animal experimentation committee (approval numbers: Med Kyo 20005, 21066).

### Maintenance of medaka

Medaka were maintained in a homebuilt water-recirculating aquarium at 26 ± 1 °C. The photoperiod was a 14-h light and 10-h dark cycle. Medaka was fed with fish food (adult and young phase: Otohime B2, Marubeni Nisshin Feed Co., Ltd., Tokyo; juvenile phase: Hikari Plankton, Kyorin Co., Ltd., Himeji, Japan) twice a day on weekdays, once a day during weekends. The number of medaka in one 2.5 L fish tank was controlled to ≤ 10.

### Maintenance of a fish tank

Medaka were maintained in translucent plastic tanks. A black sheet was kept under the tanks. Deposited dirt on the bottom of the tank was removed once every 2 weeks. In addition, *Clithon retropictus* were put into the tank to clean algae growing on the tank surface. The tanks were changed about once every 6 months in addition when the tank walls or water surface were considerably dirty.

### Subjects

Six medaka (F97; male, 3; female, 3; Individuals #1–6) and 30 medaka (F97; male, 15; female, 15; Individuals #7–36) of the inbred Hd-rR strain were used for long term and variation and stability verification, respectively. Six medaka (male, 3; female, 3; Individuals #37–42) of the non-inbred Kyoto-Cab strain, a sub-Cab strain, were used for non-inbred verification. At the time of the first image acquisition, Individuals #1–6, Individuals #7–36, and Individuals #37–42 were 3 months, 44–45 weeks, and 10 weeks of age, respectively. After the first image acquisition, animals were kept individually.

### Image acquisition for verification

**Figure 6:**
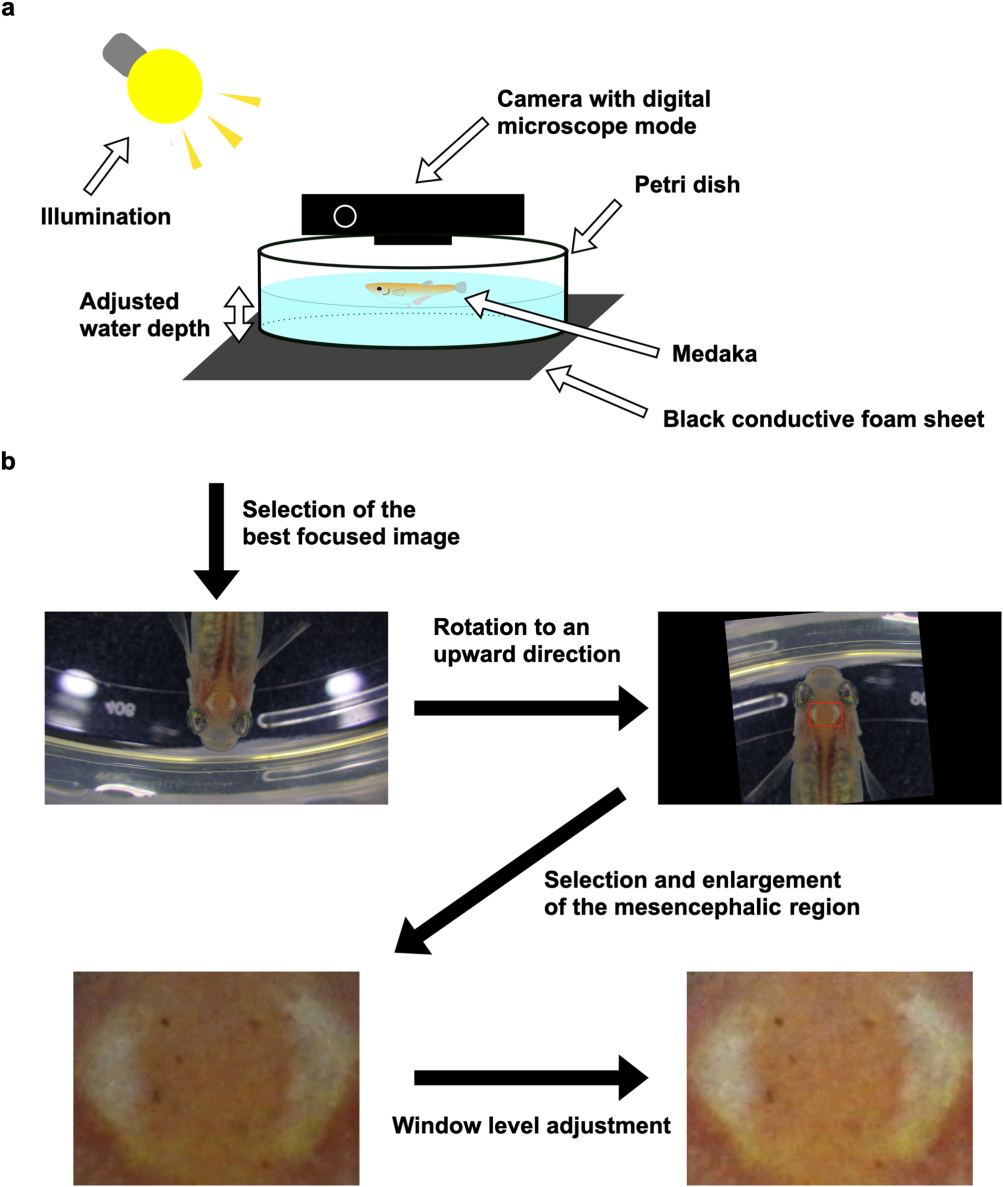
Flowchart for obtaining an individual distribution pattern of melanophore spots. (**a**) Setup for the acquisition of head images of individual medaka. A medaka was placed in a Petri dish at an adjusted water depth on a black conductive foam sheet (Supplementary Fig. 1). The illumination was provided by a ceiling and desk lights (Supplementary Fig. 2). The medaka head region was photographed from above using a camera with digital microscopic mode. (**b**) Image processing flow. The best focused image was chosen for further processing. The image was rotated to an upward position (ImageJ 1.53a) and the mesencephalic region was selected in the red rectangle (ImageJ 1.53a) and enlarged (Keynote 11.2). Finally, the window level was adjusted to facilitate melanophore spot detection (Keynote 11.2).

A single medaka fish was placed in a breeding water-filled Petri dish on a black conductive foam sheet illuminated by a ceiling and desk lights (Fig. 6a, Supplementary Figs. 1, 2). Then, the water depth was adjusted just above the fish height. When the fish rested in the water, a head image was taken from above using a digital microscope and the spot metering mode of a digital compact camera (WG-50, Ricoh Imaging Co., Ltd., Tokyo, Japan). In the digital microscope mode, the focus range was set to 0.01–0.3 m (a 1 cm Micro mode) and frame size was fixed to 1920 × 1080 pixels. A focused image showing all melanophore spots was selected from multiple images as individual identifier. If no image was suitable for further processing, we repeated the above image acquisition procedure till obtaining a suitable one.

### Image acquisition for long term verification

The sixth set of the medaka head images was taken following image acquisition for the variation and stability verification. In the first, second, and third set, a homebuilt translucent plastic container (130 mm × 115 mm × 35 mm) was used. In the fourth set, either a Petri dish or plastic container was used; in the fifth set, a Petri dish was used. Except for the sixth set, the spot metering was not used and only on-site multiple image comparison was performed.

### Image analysis

Figure 6b shows a flow chart of image analysis. First, using ImageJ 1.53a^42^, the acquired medaka head image was rotated to an upward position with a bilinear interpolation method and its mesencephalic region was manually selected in a square region of interest. Second, using Keynote (11.2, Apple Inc., California, USA), the selected region was expanded to about a 3–5 times longer size maintaining the aspect ratio for inbred and non-inbred medaka, respectively. The expansion was performed to achieve the same width in long term verification images or the same height in variation and stability, and non-inbred verification images. Finally, the window level was adjusted by visual inspection on Keynote. In images for the variation and stability verification, two red dotted lines were drawn to divide the image into four sections as a guide. Dotted lines were automatically drawn by an in-house MATLAB code (R2021a, The MathWorks, Inc., Massachusetts, USA) to divide the length and width in two equal halves. Since the mesencephalic region was manually selected, the anatomical position of the dotted line at the second time point changed from that at the first point. Therefore, dotted lines were omitted in non-inbred verification.

### Blind identification tests

Inbred and non-inbred pairing tests were performed by three examinees blinded to medaka identifiers. Examinees 1–3 had 3.5, 1.5 years, and 6 months of experience in medaka breeding, respectively. In each test, two sets of images were presented to the examinees (Supplementary Files 1, 2). In each set, the images were placed in a randomized order. Then, the examinees were instructed to match the images in the first set with those in the second set. For the inbred test, the examinees were informed that there were only 27 images in the first set (the second time point) due to death of three medaka and that the first image from the top and the first from the left in the first set should be treated as demised due to Matsukasa disease. At the end of the tests, all answers were reviewed for correspondence and the rate of agreement quantified.

## Supplementary Information

Supplementary Information 1.

Supplementary Information 2.

Supplementary Information 3.

## Data Availability

The datasets obtained in this study are available from the corresponding authors upon reasonable request.
