# Supplementary material for: Individual identification of inbred medaka based on characteristic melanophore spot patterns on the head
Source: Sci Rep. 2023 Jan 12;13:659. doi: 10.1038/s41598-023-27386-w (PMC9837133; doi:10.1038/s41598-023-27386-w)
Supplement: Supplementary file 1 — Supplementary Information 1. [file 41598_2023_27386_MOESM1_ESM.pptx]

## Slide 1
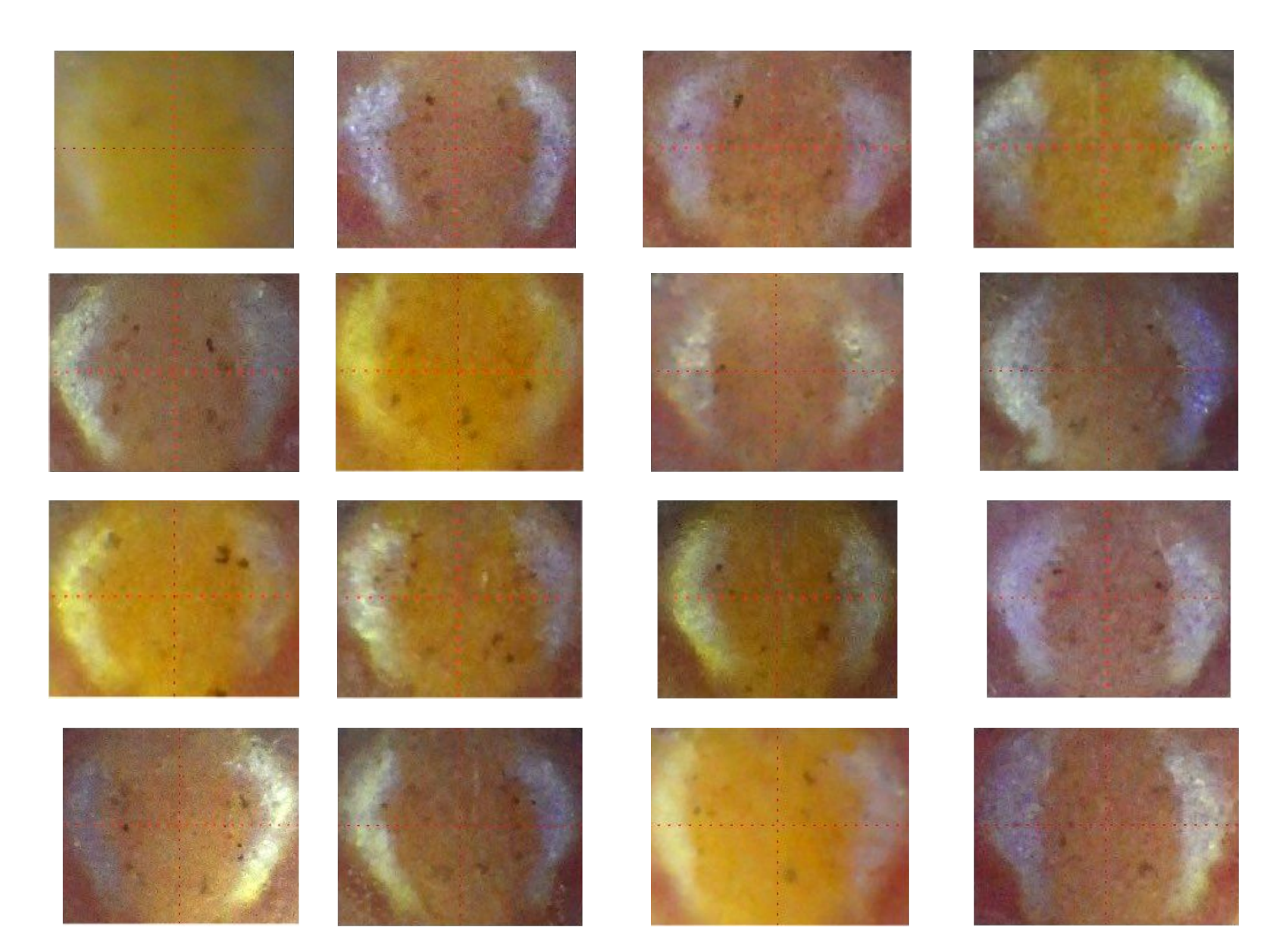

## Slide 2
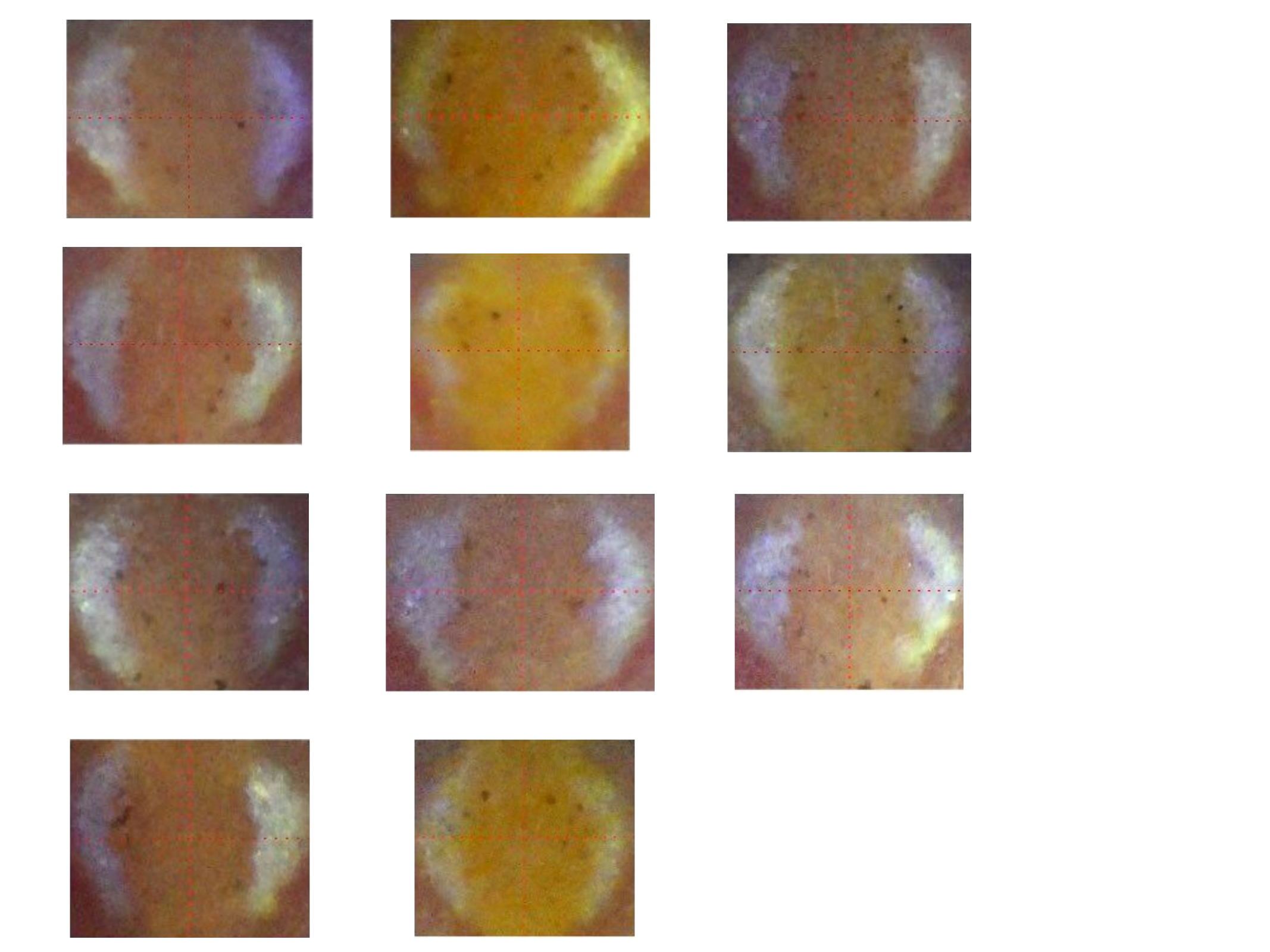

## Slide 3
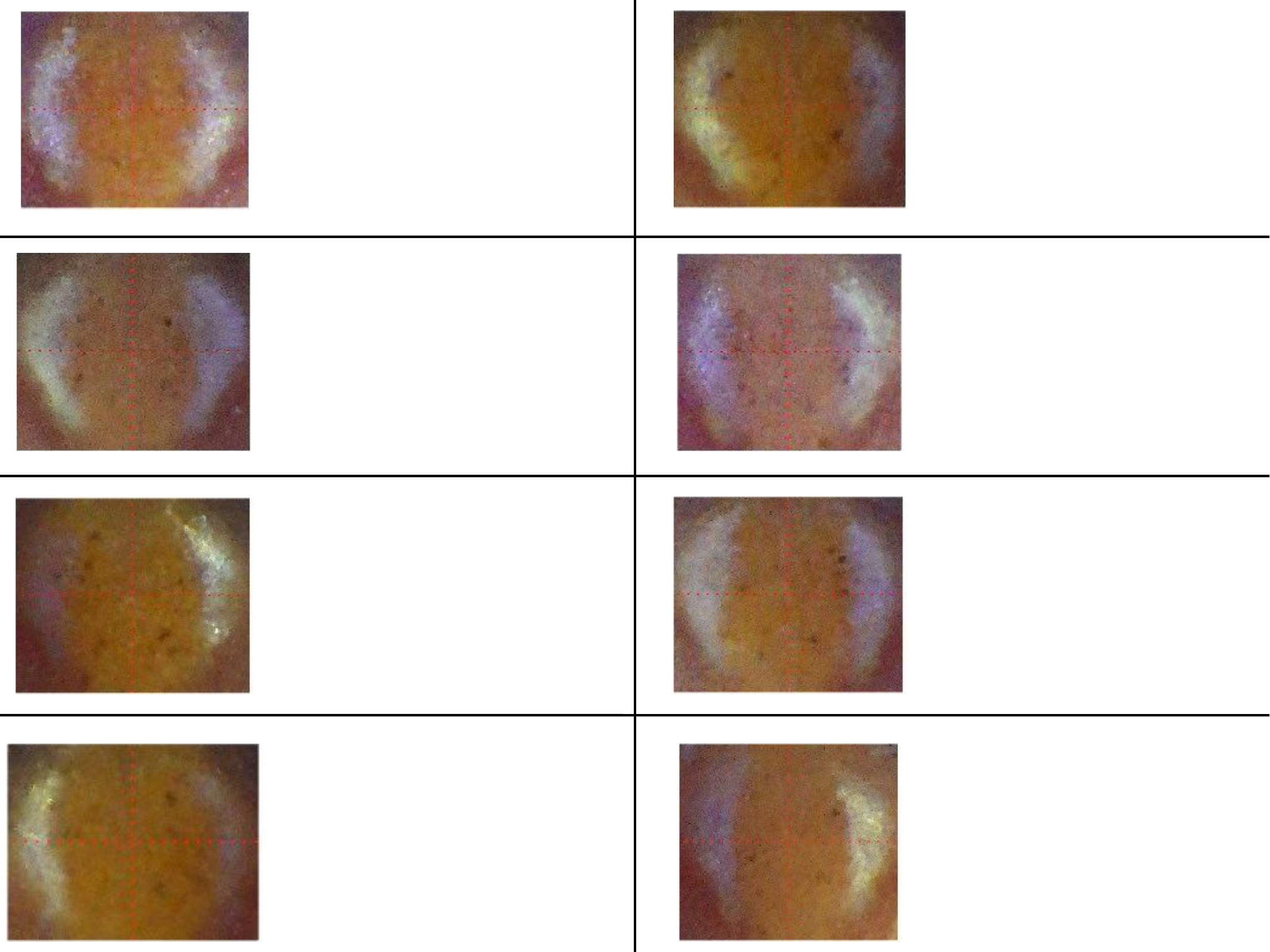

## Slide 4
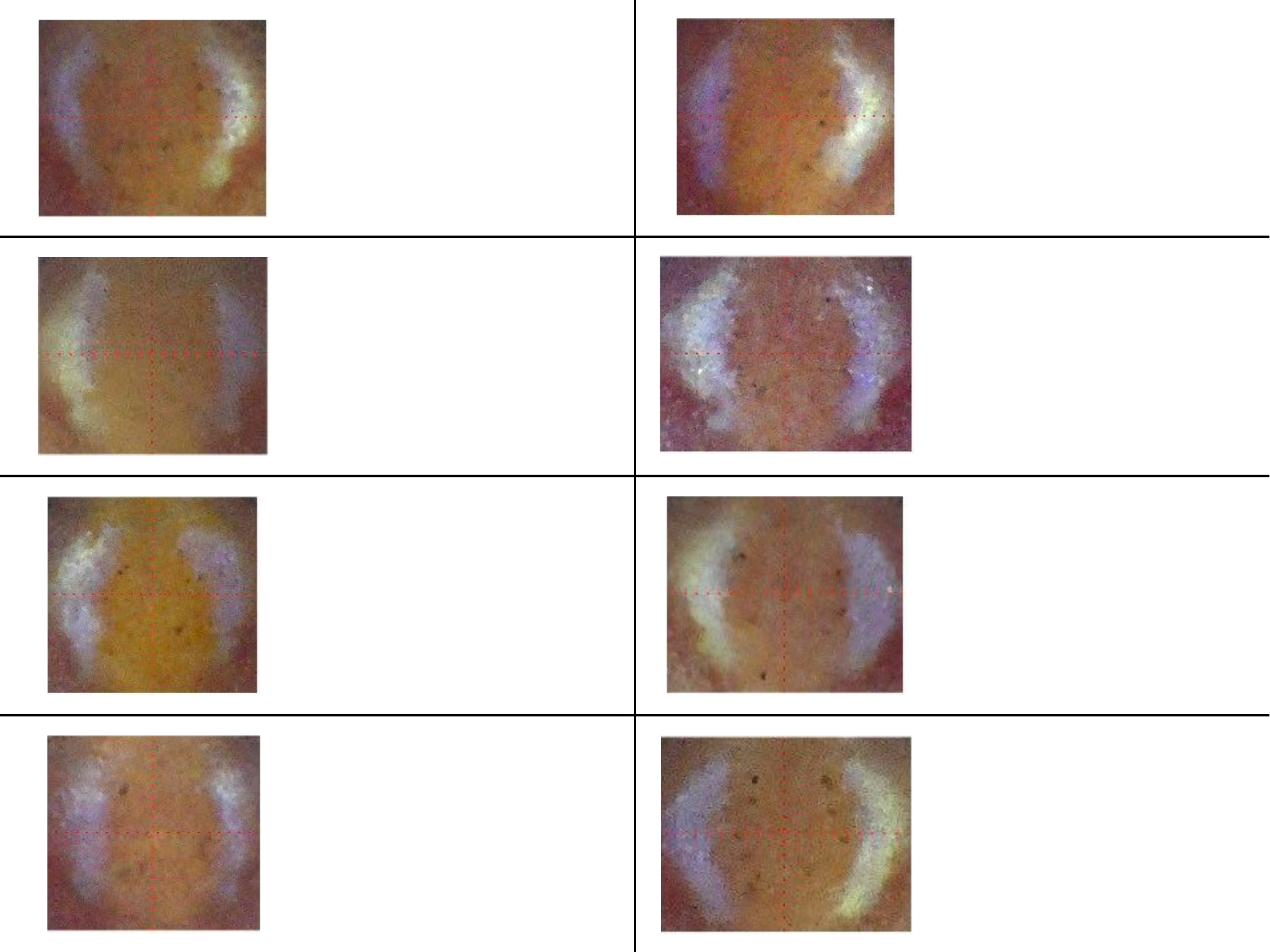

## Slide 5
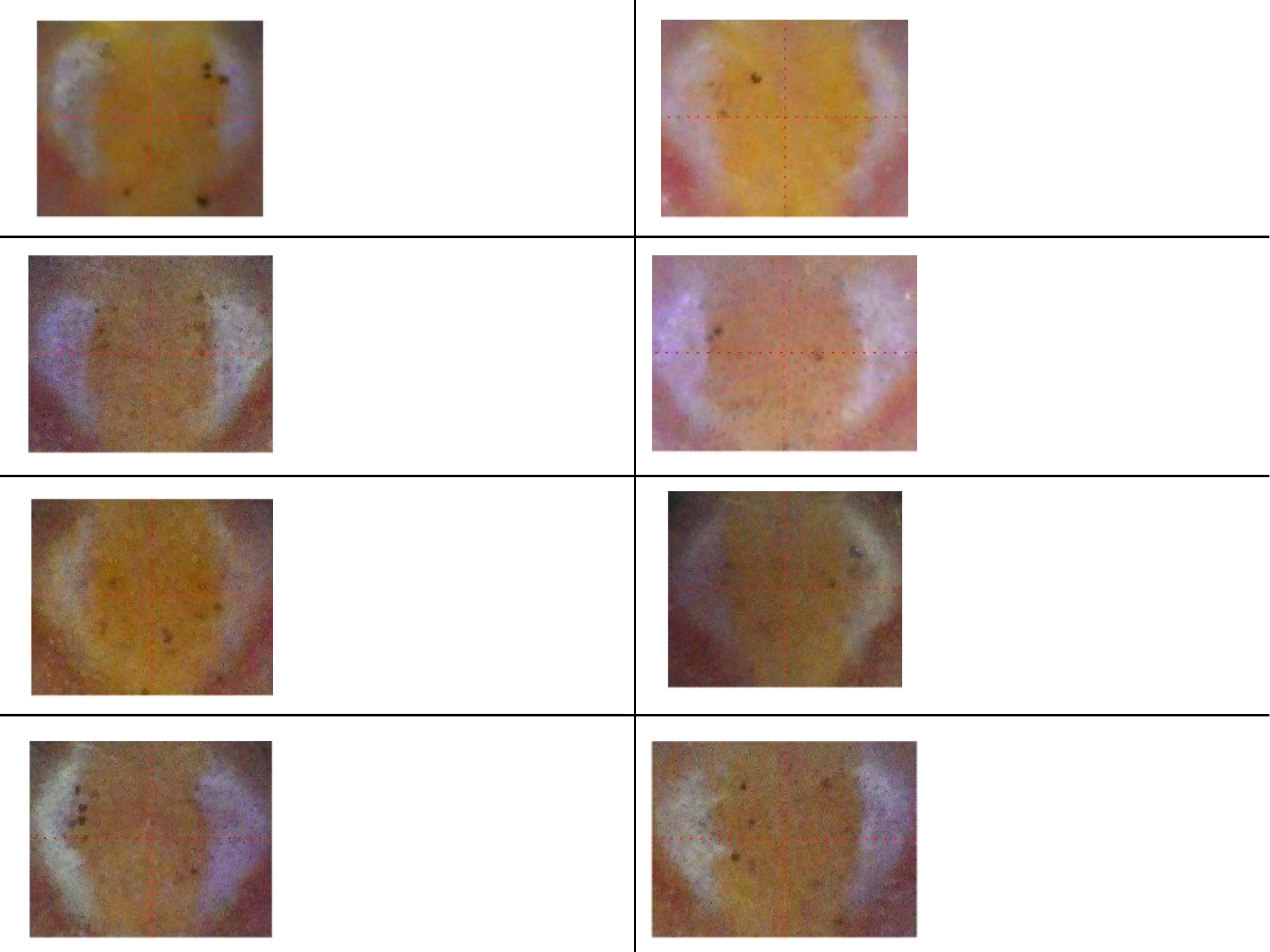

## Slide 6
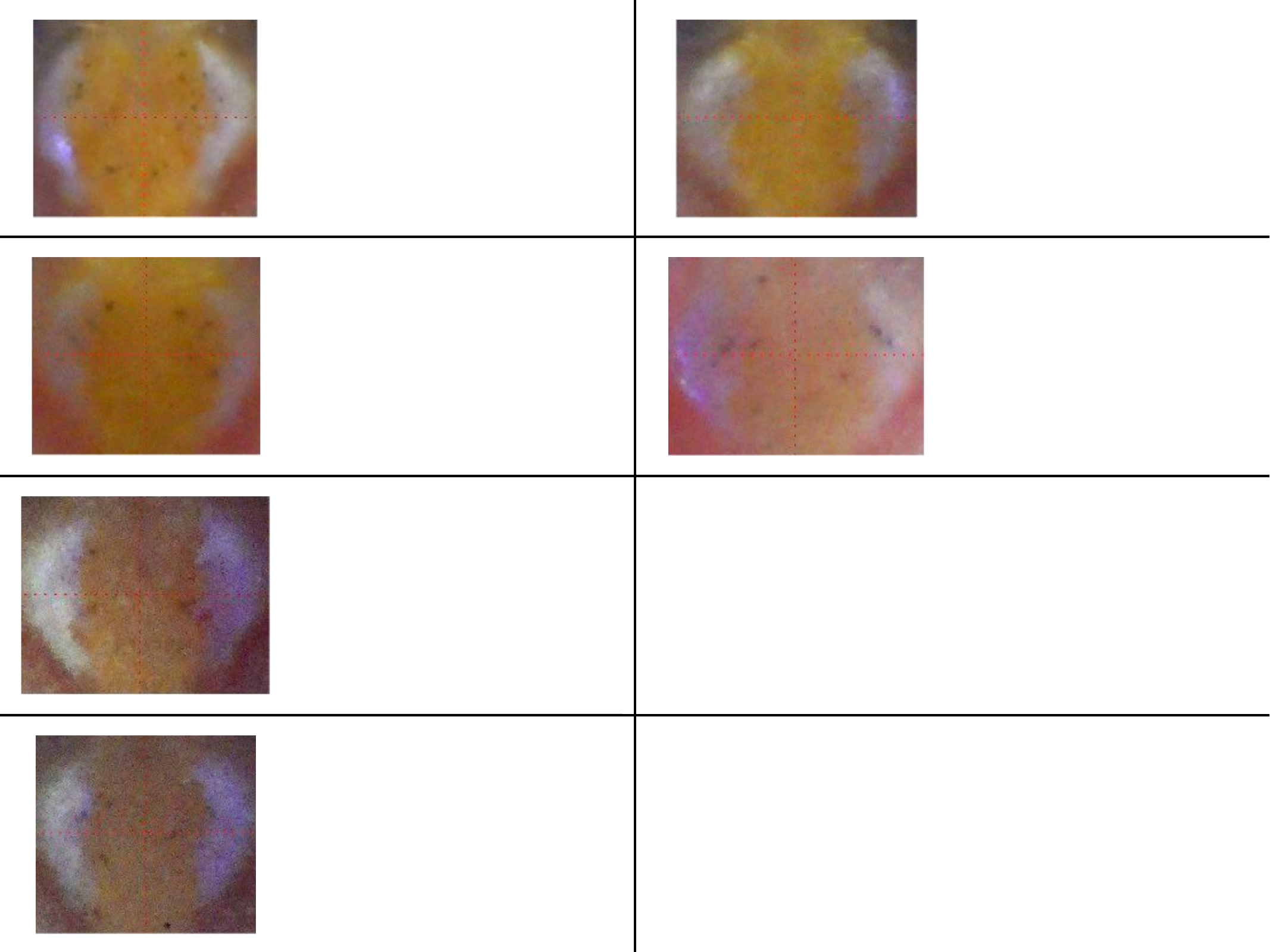

## Slide 7
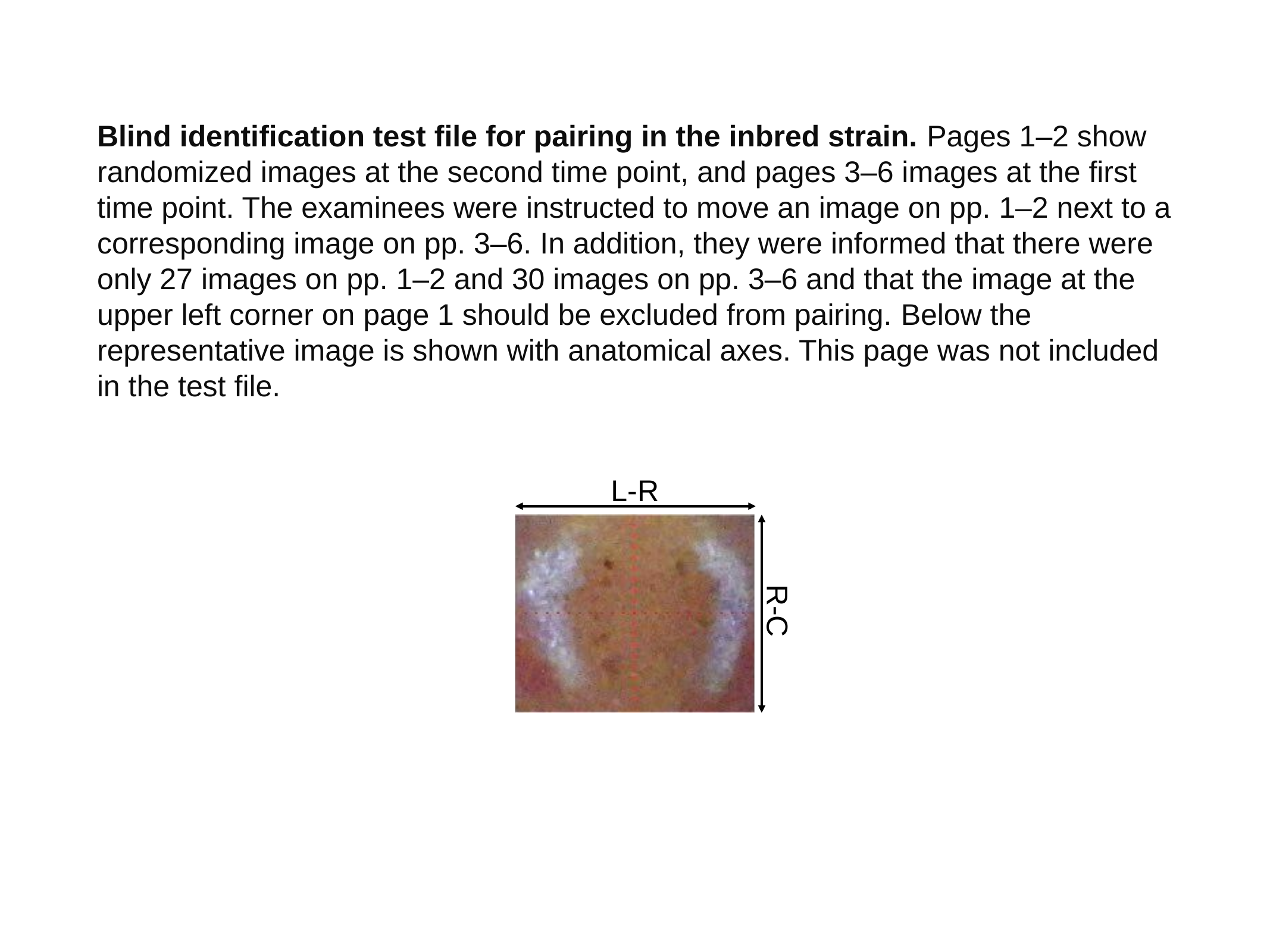

Blind identification test file for pairing in the inbred strain. Pages 1–2 show randomized images at the second time point, and pages 3–6 images at the first time point. The examinees were instructed to move an image on pp. 1–2 next to a corresponding image on pp. 3–6. In addition, they were informed that there were only 27 images on pp. 1–2 and 30 images on pp. 3–6 and that the image at the upper left corner on page 1 should be excluded from pairing. Below the representative image is shown with anatomical axes. This page was not included in the test file.
L-R
R-C
