# Supplementary material for: Individual identification of inbred medaka based on characteristic melanophore spot patterns on the head
Source: Sci Rep. 2023 Jan 12;13:659. doi: 10.1038/s41598-023-27386-w (PMC9837133; doi:10.1038/s41598-023-27386-w)
Supplement: Supplementary file 2 — Supplementary Information 2. [file 41598_2023_27386_MOESM2_ESM.pptx]

## Slide 1
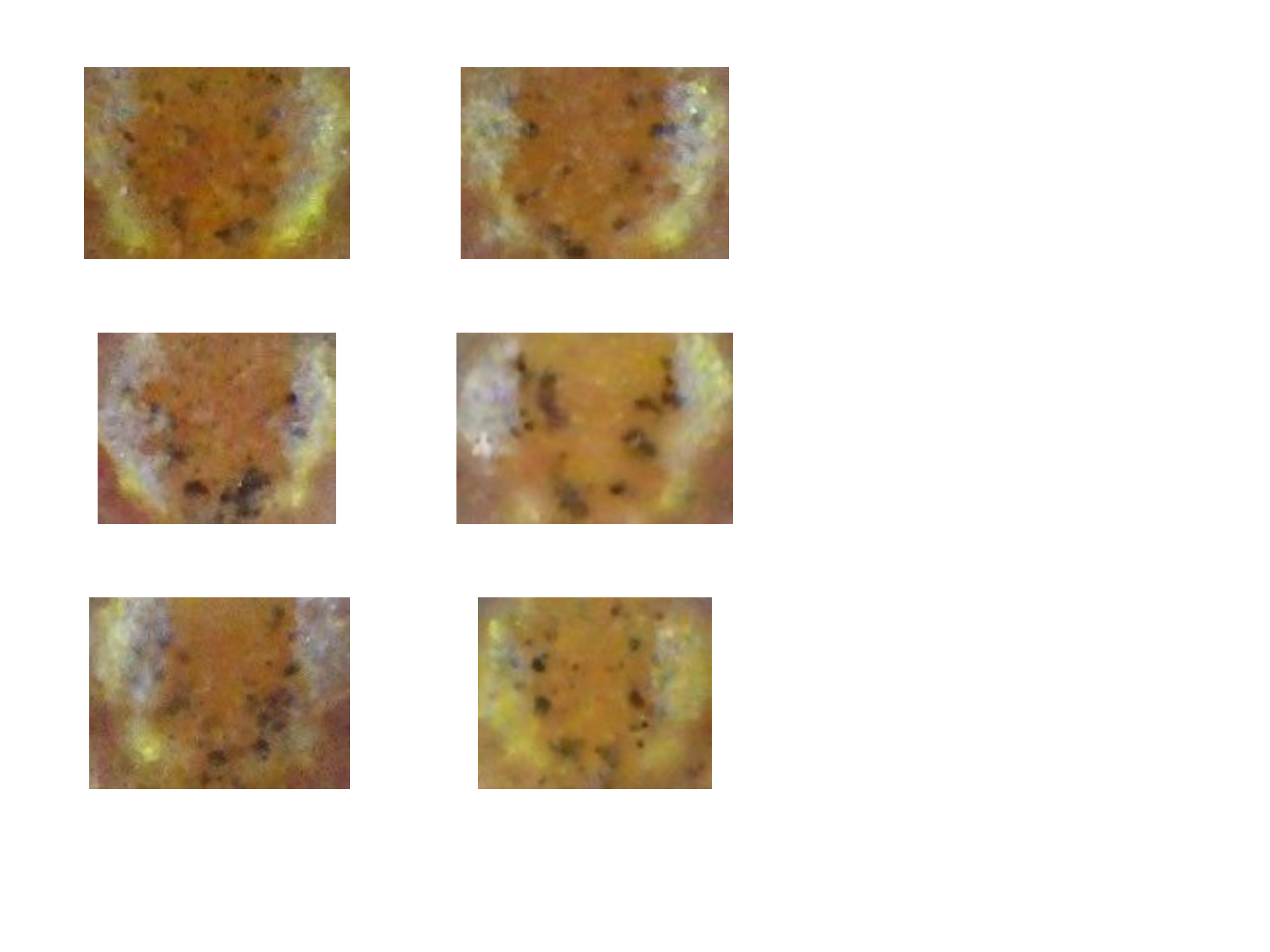

## Slide 2
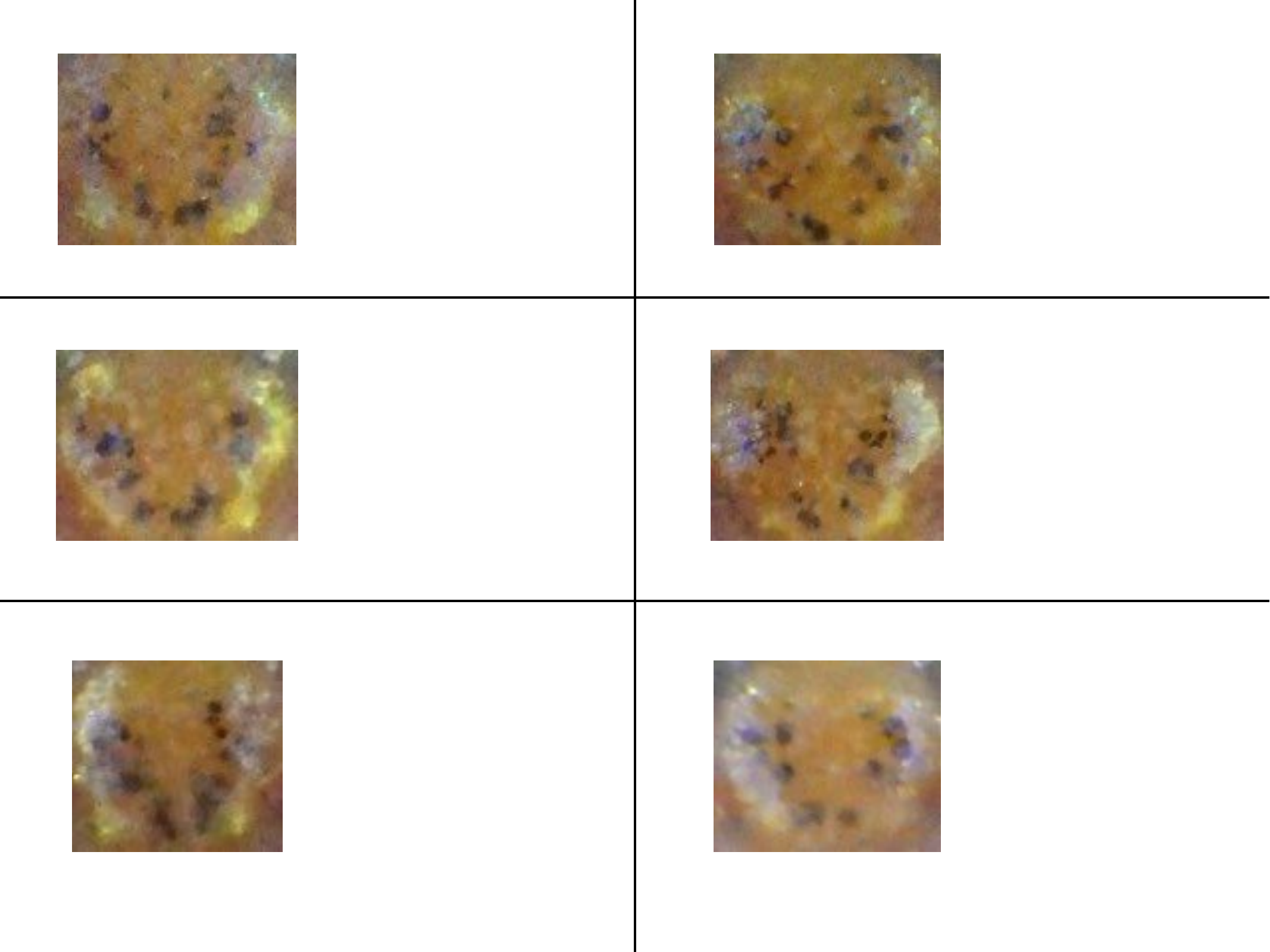

## Slide 3
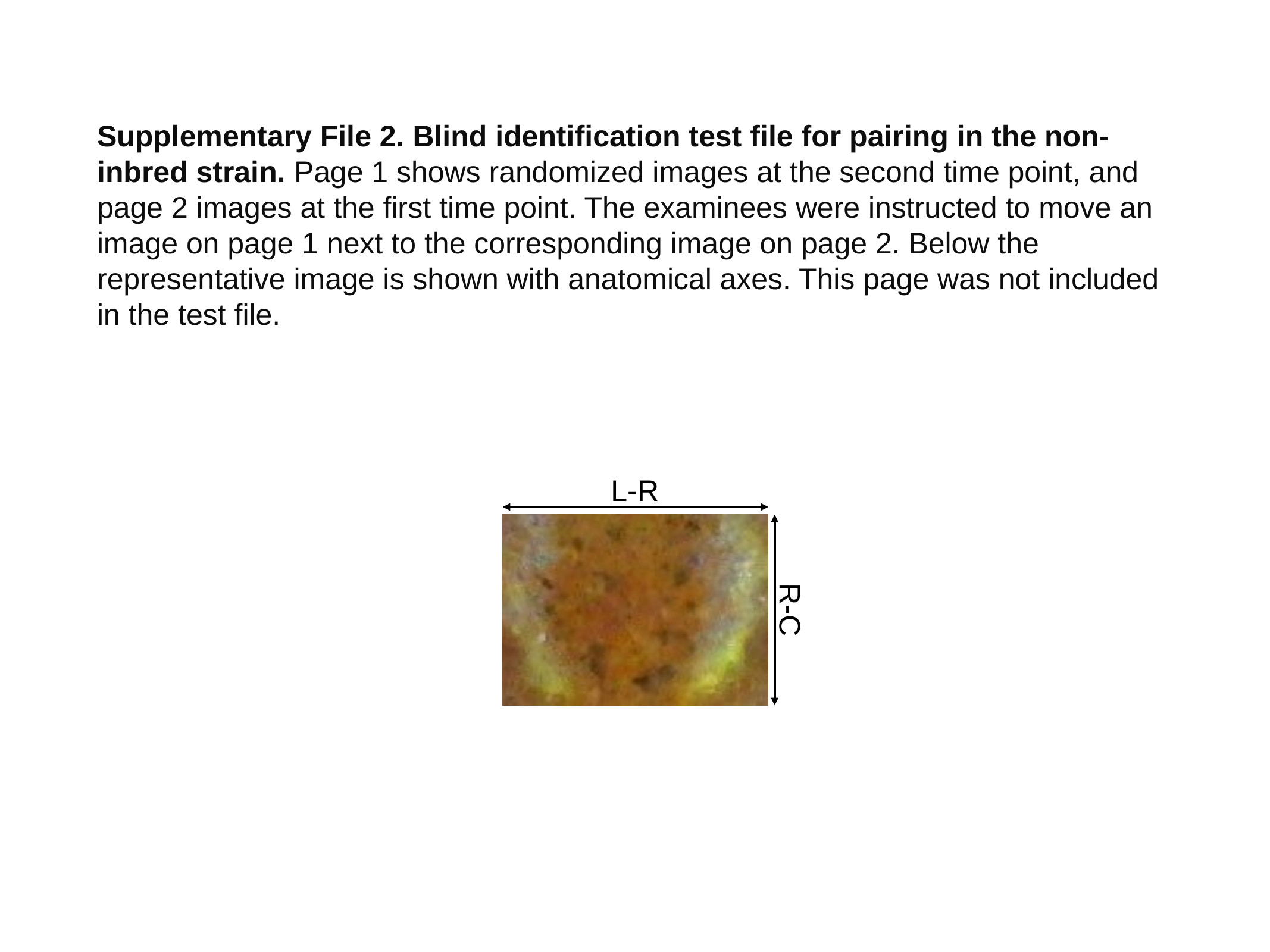

Supplementary File 2. Blind identification test file for pairing in the non-inbred strain. Page 1 shows randomized images at the second time point, and page 2 images at the first time point. The examinees were instructed to move an image on page 1 next to the corresponding image on page 2. Below the representative image is shown with anatomical axes. This page was not included in the test file.
L-R
R-C
