# Supplementary material for: Individual identification of inbred medaka based on characteristic melanophore spot patterns on the head
Source: Sci Rep. 2023 Jan 12;13:659. doi: 10.1038/s41598-023-27386-w (PMC9837133; doi:10.1038/s41598-023-27386-w)
Supplement: Supplementary file 3 — Supplementary Information 3. [file 41598_2023_27386_MOESM3_ESM.pdf]

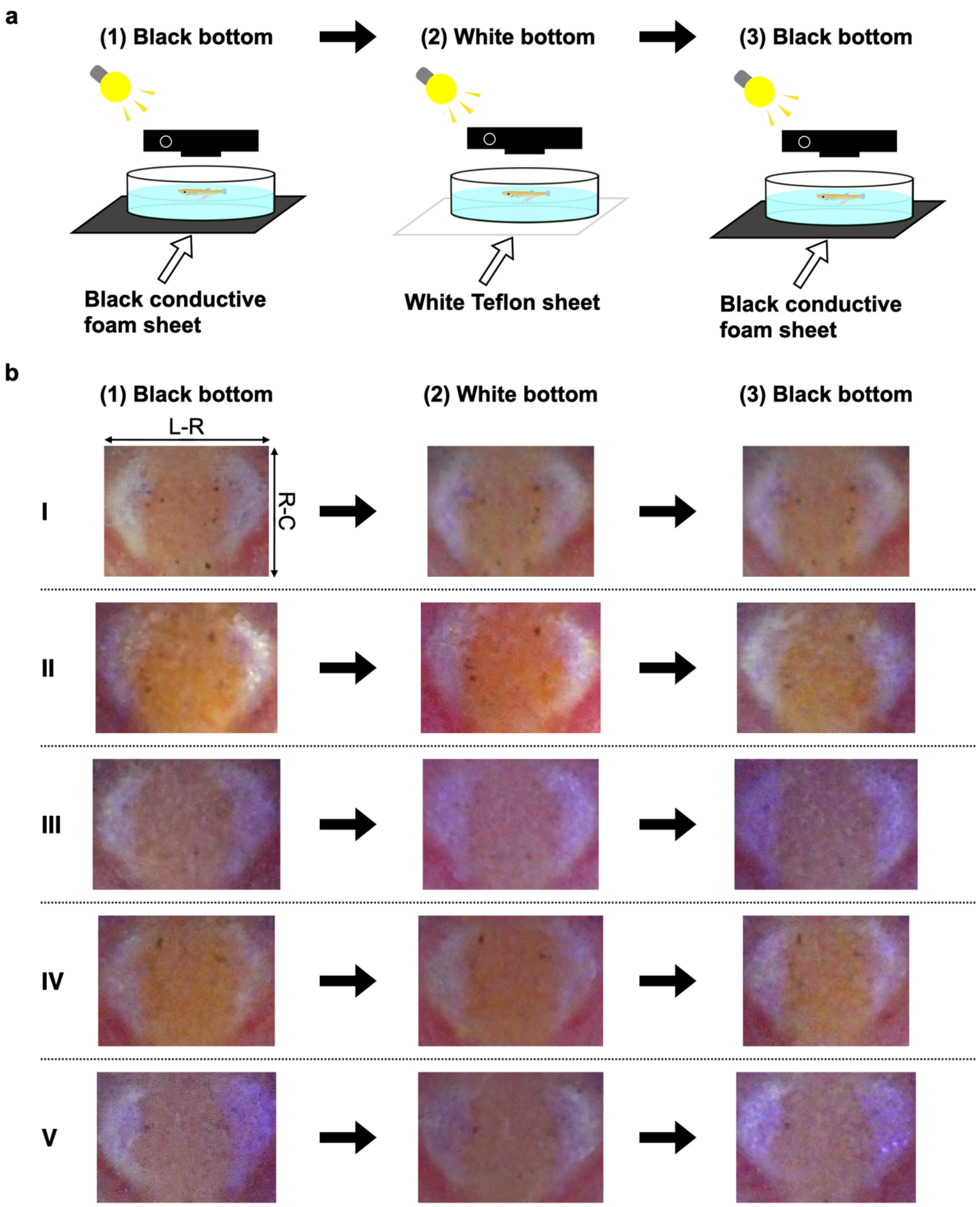

Supplementary Figure 1. A white bottom does not affect efficacy in discerning melanophore spots. (a) Flowchart of comparison between using a black or white bottom. Using a black conductive sheet or white Teflon sheet, the bottom condition in the acquisition setup (Fig. 6a) started with black (1), changed to white (2), and returned to black (3). Before acquiring the distribution patterns, the same bottom condition was maintained for ≥5 min. Only on-site image comparisons were performed. Five inbred medaka (the Hd-rR strain, F97) were utilized (I, 35 weeks of age, male; II, 24 weeks of age, male; III, 24 weeks of age, female; IV, 24 weeks of age, male; V, 24 weeks of age, female). (b) Distribution patterns of melanophore spots in the black and white bottom condition. Each row corresponds to individual medaka, and the columns correspond to initial black (1), white (2), and final black condition (3). Despite different image quality depending on bottom conditions, no trend was found among individuals. This indicates a smaller influence of bottom conditions on image quality than that of factors such as focusing. R-C: rostral-caudal axis, L-R: left-right axis.

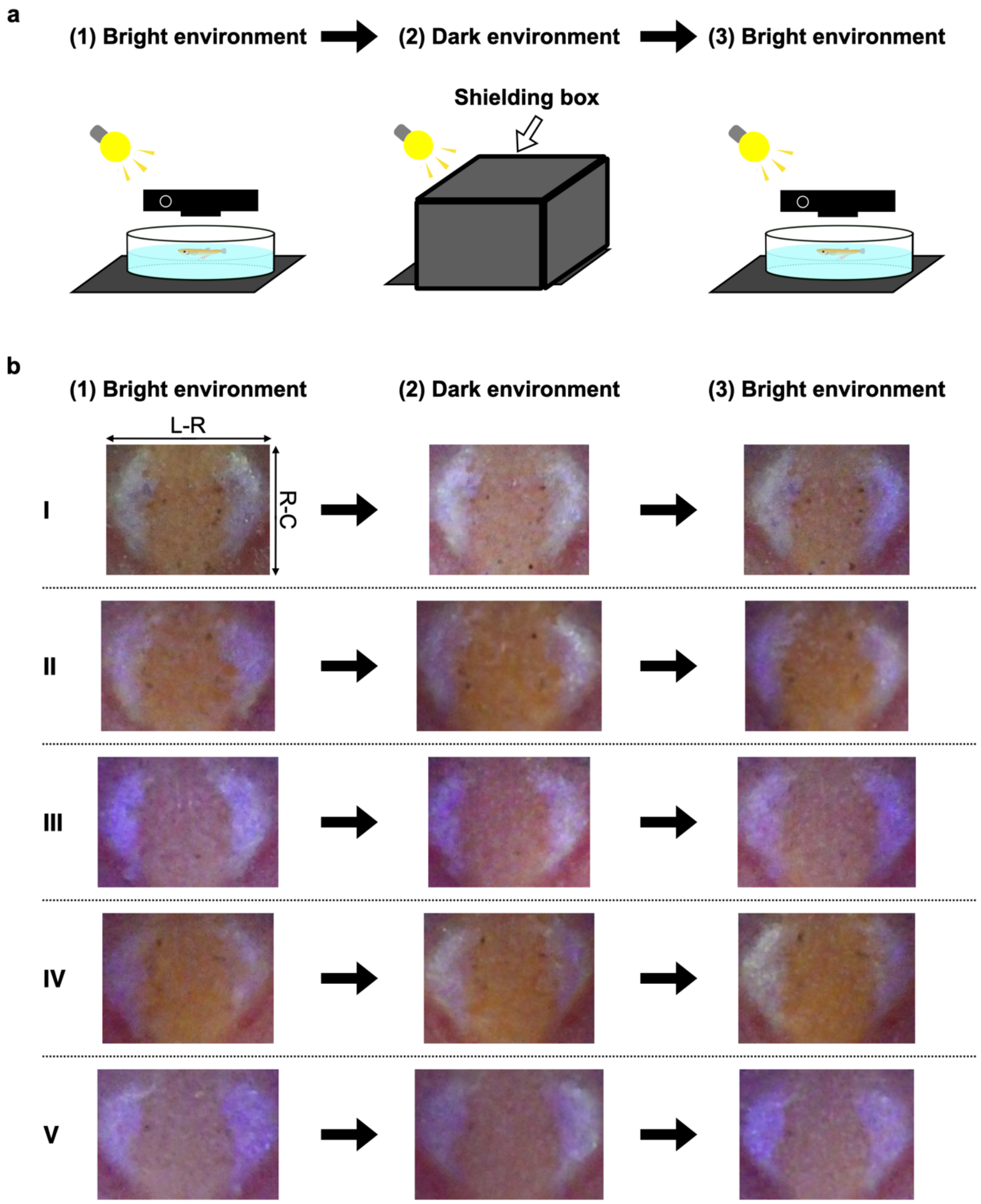

Supplementary Figure 2. A dark environment does not alter efficacy in discerning melanophore spots in a bright environment. (a) Flowchart of comparisons between bright and dark environments. The light environment in the acquisition setup (Fig. 6a) started with a bright condition (1), changed to a dark condition (2), and returned to the bright condition (3). Before acquiring distribution patterns, the same light environment was maintained for ≥5 min. The dark environment was generated by covering a Petri dish containing medaka with a shielding box, and within 30 s after removing the box, a head image was acquired. Five inbred medaka (the Hd-rR strain, F97) were utilized (I, 35 weeks of age, male; II, 24 weeks of age, male; III, 24 weeks of age, female; IV, 24 weeks of age, male; V, 24 weeks of age, female). (b) Distribution patterns of melanophore spots in the bright and dark environment. Each row corresponds to individual medaka and the columns correspond to the initial bright (1), dark (2), and final bright condition (3). Despite different image quality depending on the environmental light, no trend was found among individuals, indicating that the light environment does not have a large impact on image quality. R-C: rostral-caudal axis, L-R: left-right axis.
